# Supplementary material for: Transcriptomic and open chromatin atlas of high-resolution anatomical regions in the rhesus macaque brain
Source: Nat Commun. 2020 Jan 24;11:474. doi: 10.1038/s41467-020-14368-z (PMC6981234; doi:10.1038/s41467-020-14368-z)
Supplement: Supplementary file 1 — Supplementary Information [file 41467_2020_14368_MOESM1_ESM.pdf]

Supplementary Information

Transcriptomic and open chromatin atlas of high-resolution anatomical regions in the rhesus macaque brain

Yin et al.

Supplementary Note 1: RNA-seq quality control information

Only about 1% of the reads were ribosomal RNA (rRNA, ranged from 0.57% to 1.35%), indicating sufficient rRNA depletion. Only two samples had an RNA integrity number (RIN) of less than 5, both with high median transcript integrity number (medTIN >70, see methods, Supplementary Figure 1c). The number of expressed genes (log_2(RPKM+1)>1 or 10) in each sample had no correlation with RIN of the sample (Supplementary Figure 1d).

Supplementary Note 2: Overviewing the RNA-seq data using t-SNE

The samples from non-cortical regions, like the pons (PON) in the brainstem, clustered by regions. Whereas samples from cerebral cortical regions clustered by individuals. Similar patterns could be seen using various random seed and perplexity parameters of t-SNE (Supplementary Figure 1f-g).

Supplementary Note 3: Assembly control of novel transcripts

We compared the performance of different minimum expression cut-off values using F1 score (factor that reflected both sensitivity and precision, see methods). Maximal F1 score could be achieved at exon level when minimal expression filter set to 6.5 and maximal F1 score at intron level achieved when minimal expression filter set to 4.5 (Supplementary Figure 8a-c). Therefore, we chose minimal expression requirement of 5.5 to assemble the transcriptome with the best possible precision and sensitivity.

Supplementary Note 4: Conservation score of novel transcripts

In order to acquire cross-species conservation of novel transcripts sequences, we downloaded conservation scores from ensembl website (54_amniotes_gerp_conservation_scores.macaca_mulatta.Mmul_8.0.1.bw and 91_mammals_gerp_conservation_scores.macaca_mulatta.Mmul_8.0.1.bw). In these records, positive scores represent highly-conserved positions while negative scores represent highly-variable positions. The bigWigAverageOverBed (v2) was used to compute the average score of each

transcript. We found that most of the predicted non-coding transcripts had close to zero or negative conservation scores, indicating low conservation across the species (Supplementary Figure 8d, e). Meanwhile nearly half of the predicted coding sequence had positive conservation scores. These results suggested that the predicted coding sequences were more conserved across the species and likely to be functional.

Supplementary Note 5: Primers

The PCR primer sequences used in this study were:

1. Primers for RNA-Seq, First strand cDNA synthesis:

Random Primers 5'-NNNNNN-3'

2. Primers for RNA-Seq, PCR Library Enrichment

NEBNext Index 1 Primer for Illumina

5´-CAAGCAGAAGACGGCATACGAGATCGTGATGT

GACTGGAGTTCAGACGTGTGCTCTTCCGATC-s-T-3´

NEBNext Index 2 Primer for Illumina

5´-CAAGCAGAAGACGGCATACGAGATACATCGGT

GACTGGAGTTCAGACGTGTGCTCTTCCGATC-s-T-3´

NEBNext Index 3 Primer for Illumina

5´-CAAGCAGAAGACGGCATACGAGATGCCTAAGT

GACTGGAGTTCAGACGTGTGCTCTTCCGATC-s-T-3´

NEBNext Index 4 Primer for Illumina

5´-CAAGCAGAAGACGGCATACGAGATTGGTCAGT

GACTGGAGTTCAGACGTGTGCTCTTCCGATC-s-T-3´

NEBNext Index 5 Primer for Illumina

5´-CAAGCAGAAGACGGCATACGAGATCACTGTGT

GACTGGAGTTCAGACGTGTGCTCTTCCGATC-s-T-3´

NEBNext Index 6 Primer for Illumina

5´-CAAGCAGAAGACGGCATACGAGATATTGGCGT

GACTGGAGTTCAGACGTGTGCTCTTCCGATC-s-T-3´

NEBNext Index 7 Primer for Illumina

5´-CAAGCAGAAGACGGCATACGAGATGATCTGGT

GACTGGAGTTCAGACGTGTGCTCTTCCGATC-s-T-3´

NEBNext Index 8 Primer for Illumina
5´-CAAGCAGAAGACGGCATACGAGATTCAAGTGT
GACTGGAGTTCAGACGTGTGCTCTTCCGATC-s-T-3´
NEBNext Index 9 Primer for Illumina
5´-CAAGCAGAAGACGGCATACGAGATCTGATCGT
GACTGGAGTTCAGACGTGTGCTCTTCCGATC-s-T-3´
NEBNext Index 10 Primer for Illumina
5´-CAAGCAGAAGACGGCATACGAGATAAGCTAGT
GACTGGAGTTCAGACGTGTGCTCTTCCGATC-s-T-3´
NEBNext Index 11 Primer for Illumina
5´-CAAGCAGAAGACGGCATACGAGATGTAGCCGT
GACTGGAGTTCAGACGTGTGCTCTTCCGATC-s-T-3´
NEBNext Index 12 Primer for Illumina
5´-CAAGCAGAAGACGGCATACGAGATTACAAGGT
GACTGGAGTTCAGACGTGTGCTCTTCCGATC-s-T-3´
NEBNext Universal PCR Primer for Illumina
5´-AATGATACGGCGACCACCGAGATCTACA
CTCTTTCCCTACACGACGCTCTTCCGATC-s-T-3´
(Where -s- indicates phosphorothioate bond)

3. Primers for ATAC-Seq, Transposed DNA fragments amplification

Custom Nextera PCR Primer 1
5'-AATGATACGGCGACCACCGAGATCTACACTCGTCGGCAGCGTCAGATGTG-3'
Custom Nextera PCR Primer 2.1 (Contains Barcode)
5'-CAAGCAGAAGACGGCATACGAGATTCGCCTTAGTCTCGTGGGCTCGGAGATGT-3'
Custom Nextera PCR Primer 2.2 (Contains Barcode)
5'-CAAGCAGAAGACGGCATACGAGATCTAGTACGGTCTCGTGGGCTCGGAGATGT-3'
Custom Nextera PCR Primer 2.3 (Contains Barcode)
5'-CAAGCAGAAGACGGCATACGAGATTTCTGCCTGTCTCGTGGGCTCGGAGATGT-3'
Custom Nextera PCR Primer 2.4 (Contains Barcode)
5'-CAAGCAGAAGACGGCATACGAGATGCTCAGGAGTCTCGTGGGCTCGGAGATGT-3'
Custom Nextera PCR Primer 2.5 (Contains Barcode)
5'-CAAGCAGAAGACGGCATACGAGATAGGAGTCCGTCTCGTGGGCTCGGAGATGT-3'
Custom Nextera PCR Primer 2.6 (Contains Barcode)
5'-CAAGCAGAAGACGGCATACGAGATCATGCCTAGTCTCGTGGGCTCGGAGATGT-3'
Custom Nextera PCR Primer 2.7 (Contains Barcode)
5'-CAAGCAGAAGACGGCATACGAGATGTAGAGAGGTCTCGTGGGCTCGGAGATGT-3'

Custom Nextera PCR Primer 2.8 (Contains Barcode)
5'-CAAGCAGAAGACGGCATACGAGATCCTCTCTGGTCTCGTGGGCTCGGAGATGT-3'
Custom Nextera PCR Primer 2.9 (Contains Barcode)
5'-CAAGCAGAAGACGGCATACGAGATAGCGTAGCGTCTCGTGGGCTCGGAGATGT-3'
Custom Nextera PCR Primer 2.10 (Contains Barcode)
5'-CAAGCAGAAGACGGCATACGAGATCAGCCTCGGTCTCGTGGGCTCGGAGATGT-3'
Custom Nextera PCR Primer 2.11 (Contains Barcode)
5'-CAAGCAGAAGACGGCATACGAGATTGCCTCTTGTCTCGTGGGCTCGGAGATGT-3'
Custom Nextera PCR Primer 2.12 (Contains Barcode)
5'-CAAGCAGAAGACGGCATACGAGATTCCTCTACGTCTCGTGGGCTCGGAGATGT-3'
Custom Nextera PCR Primer 2.13 (Contains Barcode)
5'-CAAGCAGAAGACGGCATACGAGATATCACGACGTCTCGTGGGCTCGGAGATGT-3'
Custom Nextera PCR Primer 2.14 (Contains Barcode)
5'-CAAGCAGAAGACGGCATACGAGATACAGTGGTGTCTCGTGGGCTCGGAGATGT-3'
Custom Nextera PCR Primer 2.15 (Contains Barcode)
5'-CAAGCAGAAGACGGCATACGAGATCAGATCCAGTCTCGTGGGCTCGGAGATGT-3'
Custom Nextera PCR Primer 2.16 (Contains Barcode)
5'-CAAGCAGAAGACGGCATACGAGATACAAACGGGTCTCGTGGGCTCGGAGATGT-3'
Custom Nextera PCR Primer 2.17 (Contains Barcode)
5'-CAAGCAGAAGACGGCATACGAGATACCCAGCAGTCTCGTGGGCTCGGAGATGT-3'
Custom Nextera PCR Primer 2.18 (Contains Barcode)
5'-CAAGCAGAAGACGGCATACGAGATAACCCCTCGTCTCGTGGGCTCGGAGATGT-3'
Custom Nextera PCR Primer 2.19 (Contains Barcode)
5'-CAAGCAGAAGACGGCATACGAGATCCCAACCTGTCTCGTGGGCTCGGAGATGT-3'
Custom Nextera PCR Primer 2.20 (Contains Barcode)
5'-CAAGCAGAAGACGGCATACGAGATCACCACACGTCTCGTGGGCTCGGAGATGT-3'
Custom Nextera PCR Primer 2.21 (Contains Barcode)
5'-CAAGCAGAAGACGGCATACGAGATGAAACCCAGTCTCGTGGGCTCGGAGATGT-3'
Custom Nextera PCR Primer 2.22 (Contains Barcode)
5'-CAAGCAGAAGACGGCATACGAGATTGTGACCAGTCTCGTGGGCTCGGAGATGT-3'
Custom Nextera PCR Primer 2.23 (Contains Barcode)
5'-CAAGCAGAAGACGGCATACGAGATAGGGTCAAGTCTCGTGGGCTCGGAGATGT-3'
Custom Nextera PCR Primer 2.24 (Contains Barcode)
5'-CAAGCAGAAGACGGCATACGAGATAGGAGTGGGTCTCGTGGGCTCGGAGATGT-3'

Supplementary Figures

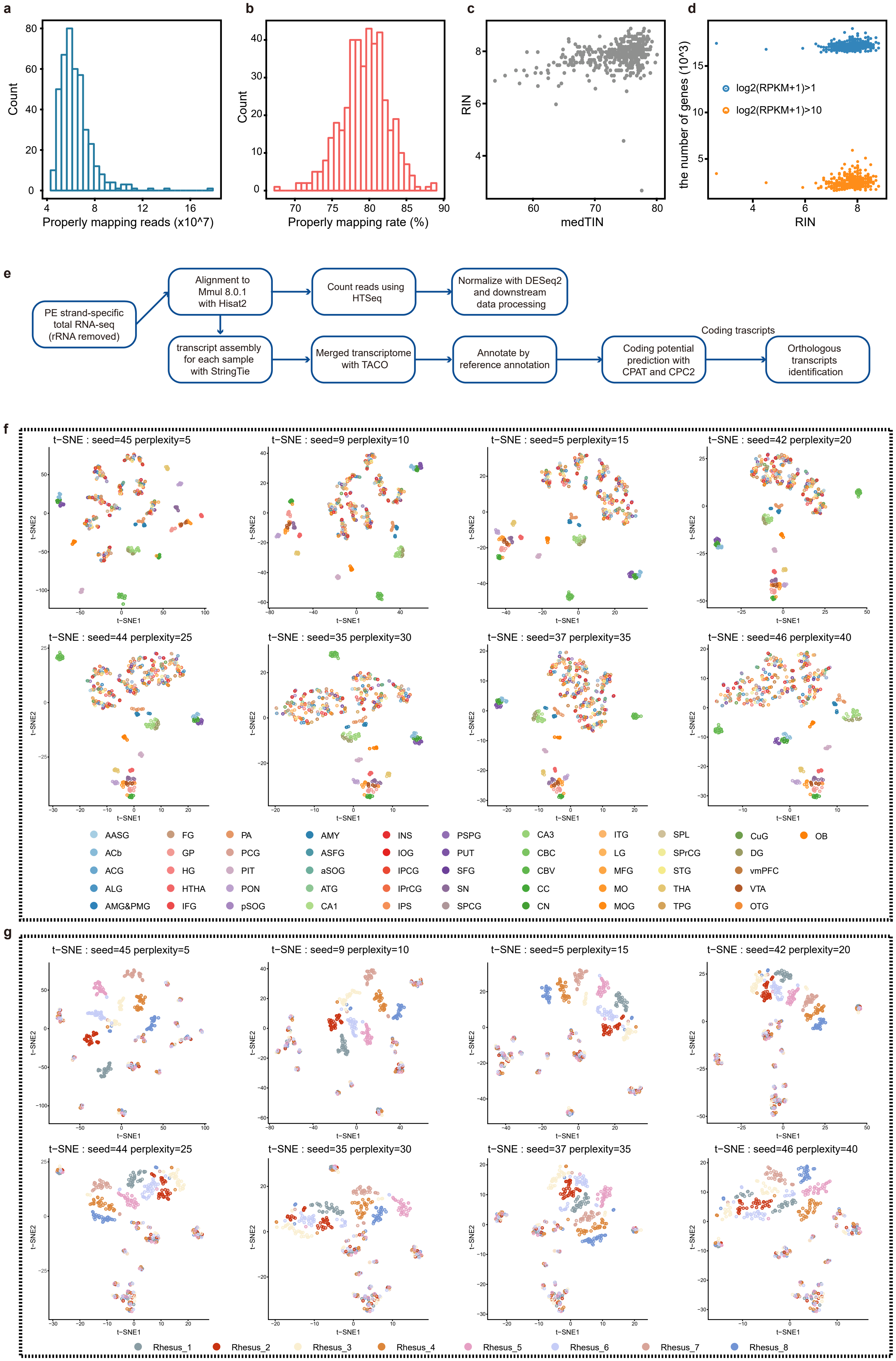

Supplementary Figure 1. RNA-seq mapping quality and analysis framework. (a) Distribution of the properly mapping reads of RNA-seq. (b) Distribution of the properly mapping rate of RNA-seq. (c) Point plot showing the correlation between medTIN (median transcript integrity number score) and RIN (RNA integrity number). (d) Point plot showing the number of genes with log2(rawRPKM+1) > 1 and log2(rawRPKM+1) > 10 against RIN. (e) The pipeline for RNA-seq data processing. (f) t-SNE scatterplot of 408 samples. The dot color represented its origin of region and perplexity setting that varied from 5 to 40. (g) t-SNE scatterplot of 408 samples. The dot color represented its origin of individual and perplexity setting that varied from 5 to 40. Source data are provided as a Source Data file.

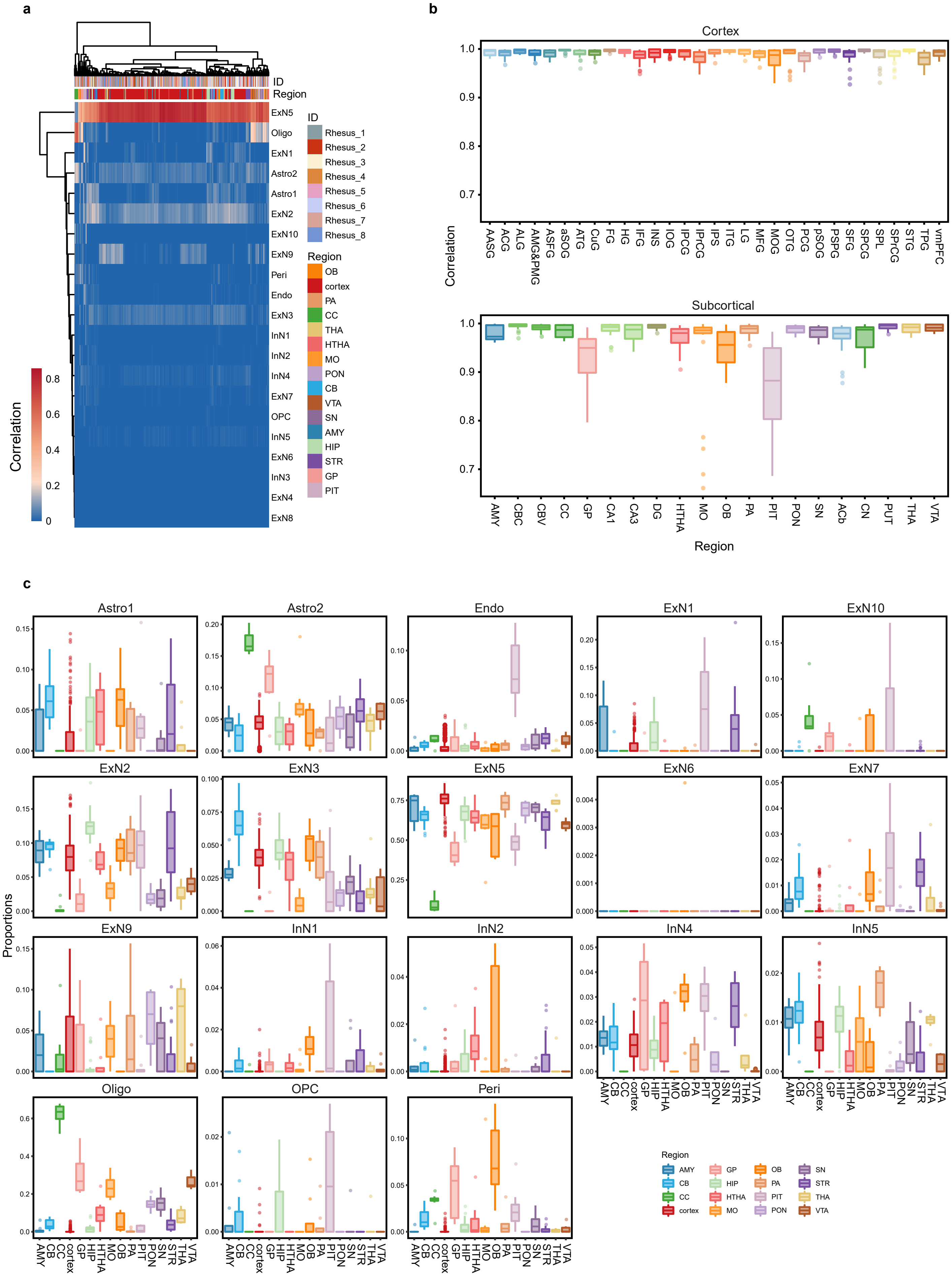

a
b
c
ID
Region
Correlation
Rhesus_1
Rhesus_2
Rhesus_3
Rhesus_4
Rhesus_5
Rhesus_6
Rhesus_7
Rhesus_8
OB
cortex
PA
CC
THA
HTHA
MO
PON
CB
VTA
SN
AMY
HIP
STR
GP
PIT
ExN5
Oligo
ExN1
Astro2
Astro1
ExN2
ExN10
ExN9
Peri
Endo
ExN3
InN1
InN2
InN4
ExN7
OPC
InN5
ExN6
InN3
ExN4
ExN8
Cortex
Subcortical
AASG ACG ALG AMG&PMG ASFG aSOG ATG CuG FG HG IFG INS IOG IPCG lPCG IPS ITG LG MFG MOG OTG PCG pSOG PSPG SFG SPCG SPL SPrCG STG TPG vmPFC
AMY CBC CBV CC GP CA1 CA3 DG HTHA MO OB PA PIT PON SN ACb CN PUT THA VTA
Region
Proportions
Astro1
Astro2
Endo
ExN1
ExN10
ExN2
ExN3
ExN5
ExN6
ExN7
ExN9
InN1
InN2
InN4
InN5
Oligo
OPC
Peri
AMY CB CC cortex GP HIP HTHA MO OB PA PIT PON SN STR THA VTA
Region
AMY
CB
CC
cortex
GP
HIP
HTHA
MO
OB
PA
PIT
PON
SN
STR
THA
VTA

Supplementary Figure 2. Bulk tissue deconvolution of macaque brains. (a) Heatmap showing the proportions of different types of cells in bulk samples. (ExN for excitatory neurons, InN for interneuron, Astro for astrocyte, OPC for oligodendrocyte progenitor cell, Oligo for oligodendrocyte, Endo for endothelial cell, Peri for pericytes)**.** (b) Boxplot showing the distribution of Pearson's r between inferred cellular composition of individuals in each region (the cortex and subcortical structures). (c) Boxplot showing the distribution of proportions of a certain cell type across the brain regions.In boxplots, center line, box edges, whiskers and points indicate the median, upper and lower quartiles (the 25th and 75th percentiles), 1.5× interquartile range and outlier, respectively. Source data are provided as a Source Data file.

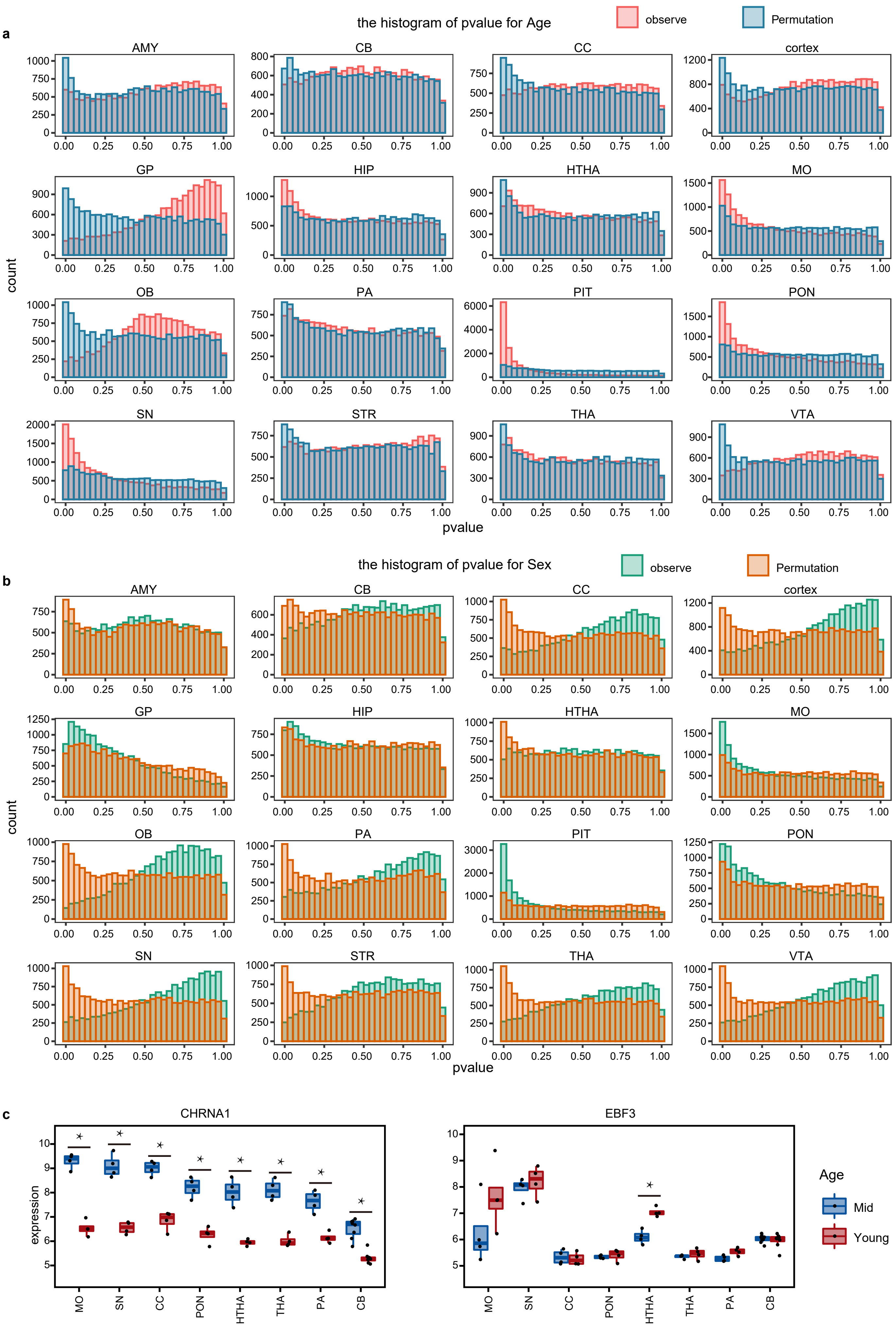
a
the histogram of pvalue for Age
observe
Permutation
AMY
CB
CC
cortex
GP
HIP
HTHA
MO
OB
PA
PIT
PON
SN
STR
THA
VTA
count
pvalue
b
the histogram of pvalue for Sex
observe
Permutation
AMY
CB
CC
cortex
GP
HIP
HTHA
MO
OB
PA
PIT
PON
SN
STR
THA
VTA
count
pvalue
c
CHRNA1
EBF3
expression
MO
SN
CC
PON
HTHA
THA
PA
CB
Age
Mid
Young

Supplementary Figure 3. Age and sex-related differentially expressed genes. (a) Histogram plot showing the distribution of observed and permutation-based p-value with differential expression analysis between young and middle in each region. (b) Histogram plot showing the distribution of observed and permutation-based p-value with differential expression analysis between male and female in each region. (c) Boxplot showing the expression of CHRNA1 and EBF3 between mid-aged and young. The asterisks indicated FDR < 0.1 in differential expression analysis between the young and mid-aged. In boxplots, center line, box edges and whiskers indicate the median, upper and lower quartiles (the 25th and 75th percentiles) and 1.5× interquartile range, respectively. Source data are provided as a Source Data file. Source data are provided as a Source Data file.

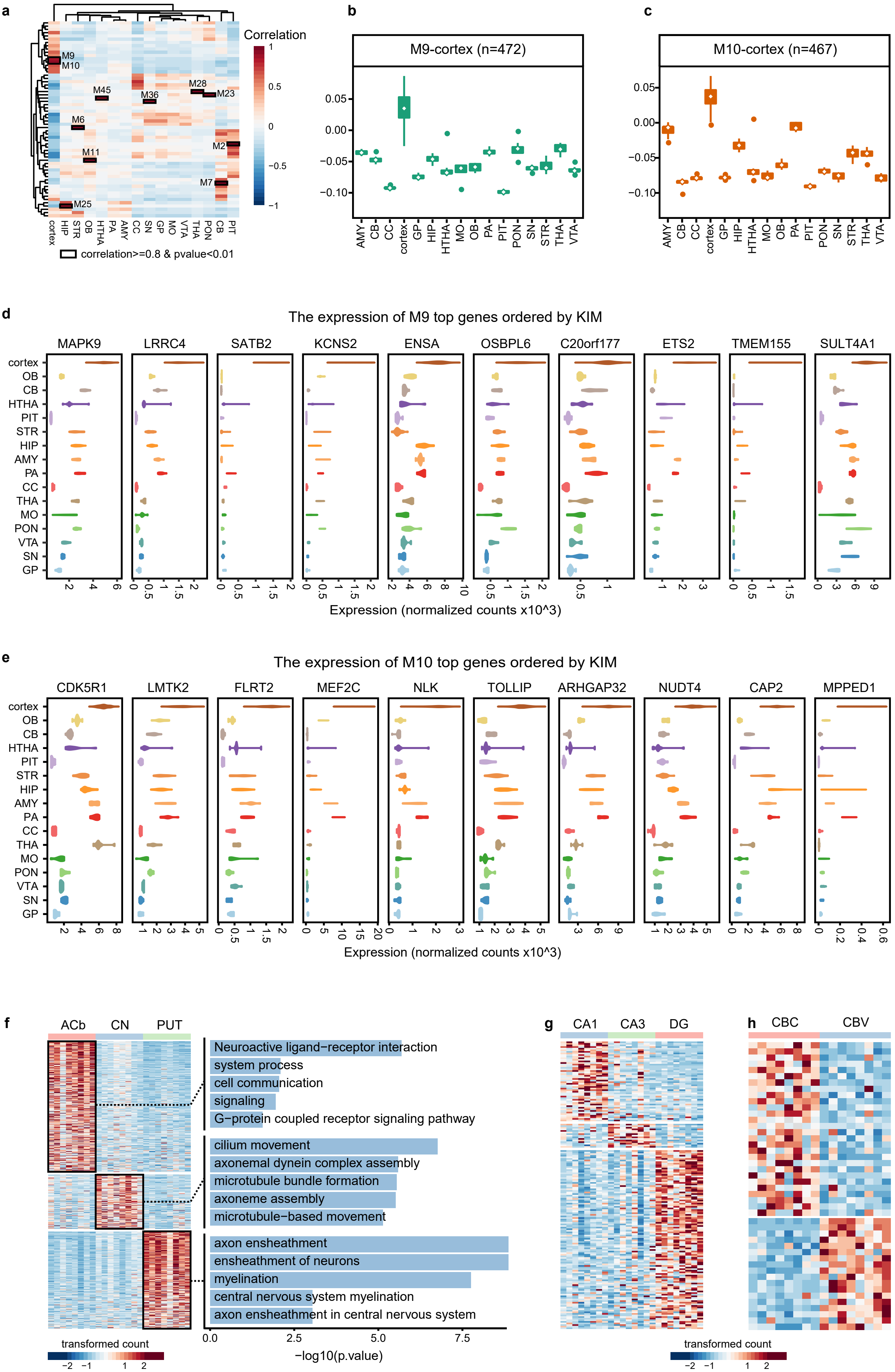
a
Correlation
M9
M10
M45
M36
M28
M23
M6
M11
M2
M7
M25
cortex HIP STR OB HTHA PA AMY CC SN GP MO VTA THA PON CB PIT
correlation>=0.8 & pvalue<0.01
b
M9-cortex (n=472)
c
M10-cortex (n=467)
AMY CB CC cortex GP HIP HTHA MO OB PA PIT PON SN STR THA VTA
d
The expression of M9 top genes ordered by KIM
MAPK9
LRRC4
SATB2
KCNS2
ENSA
OSBPL6
C20orf177
ETS2
TMEM155
SULT4A1
cortex OB CB HTHA PIT STR HIP AMY PA CC THA MO PON VTA SN GP
Expression (normalized counts x10^3)
e
The expression of M10 top genes ordered by KIM
CDK5R1
LMTK2
FLRT2
MEF2C
NLK
TOLLIP
ARHGAP32
NUDT4
CAP2
MPPED1
Expression (normalized counts x10^3)
f
ACb
CN
PUT
Neuroactive ligand−receptor interaction
system process
cell communication
signaling
G−protein coupled receptor signaling pathway
cilium movement
axonemal dynein complex assembly
microtubule bundle formation
axoneme assembly
microtubule−based movement
axon ensheathment
ensheathment of neurons
myelination
central nervous system myelination
axon ensheathment in central nervous system
−log10(p.value)
transformed count
g
CA1
CA3
DG
h
CBC
CBV
transformed count

Supplementary Figure 4. Region-specific modules. (a) Heatmap showing correlation between sub-regions and gene modules discovered using WGCNA. (b) Boxplot showing expression of the module eigengenes of the cortex specific gene module M9, the parenthesized number represented number of genes in each module. (c) Boxplot showing expression of the module eigengenes of the cortex specific gene module M10, the parenthesized number represented number of genes in each module. (d) Violin plot showing the expression of the top 10 genes in cortex-specific module M9. (e) Violin plot showing the expression of the top 10 genes in cortex-specific module M10. (f) Left: heatmap showing the expression of the subregion-specific upregulated genes in STR. Right: enriched GO-term of subregion-specific upregulated genes. (j) Heatmap showing the expression of the subregion-specific upregulated genes in HIP. (h) Heatmap showing the expression of the subregion-specific upregulated genes in CB. In boxplots, center point, box edges and whiskers indicate the median, upper and lower quartiles (the 25th and 75th percentiles) and 1.5× interquartile range, respectively. Source data are provided as a Source Data file.

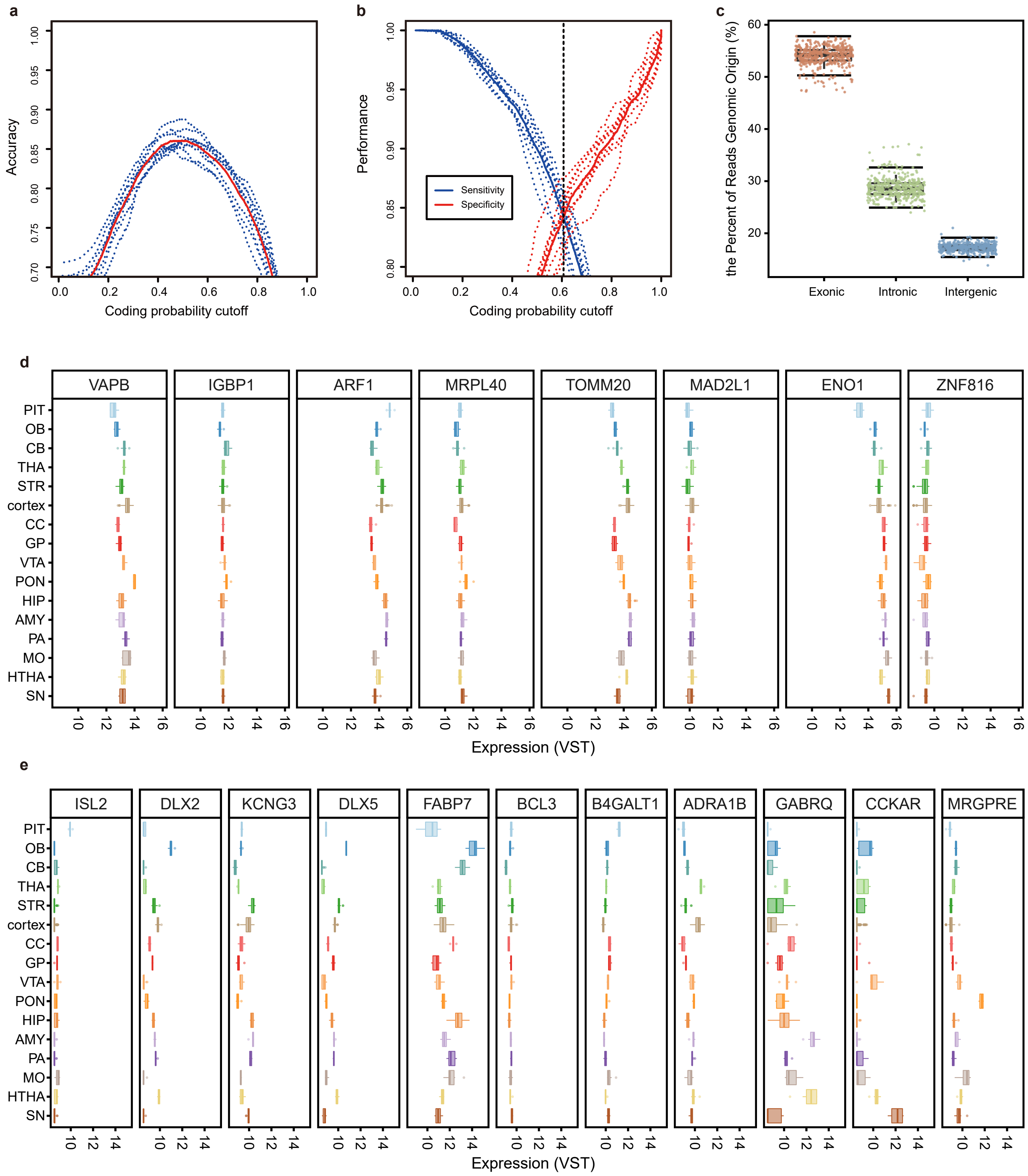

a
Accuracy
Coding probability cutoff
b
Performance
Sensitivity
Specificity
Coding probability cutoff
c
the Percent of Reads Genomic Origin (%)
Exonic
Intronic
Intergenic
d
VAPB
IGBP1
ARF1
MRPL40
TOMM20
MAD2L1
ENO1
ZNF816
PIT
OB
CB
THA
STR
cortex
CC
GP
VTA
PON
HIP
AMY
PA
MO
HTHA
SN
Expression (VST)
e
ISL2
DLX2
KCNG3
DLX5
FABP7
BCL3
B4GALT1
ADRA1B
GABRQ
CCKAR
MRGPRE
Expression (VST)

Supplementary Figure 5. Assembly cutoff and the expression of novel transcripts. (a) Accuracy versus CPAT coding probability cutoff value. (b) Two-graph ROC curve is used to determine the optimum CPAT probability cutoff value. (c) Boxplot showing the percentage of RNA-seq reads mapped to different genomic regions on the rhesus macaque assembly genome. In boxplots, center line, box edges and whiskers indicate the median, upper and lower quartiles (the 25th and 75th percentiles) and 1.5× interquartile range, respectively. (d) Violin plot showing the expression of eight novel transcripts annotated at different locations (Mmul 8.0.1.91). (e) Violin plot showing the expression of novel signature transcripts. Source data are provided as a Source Data file.

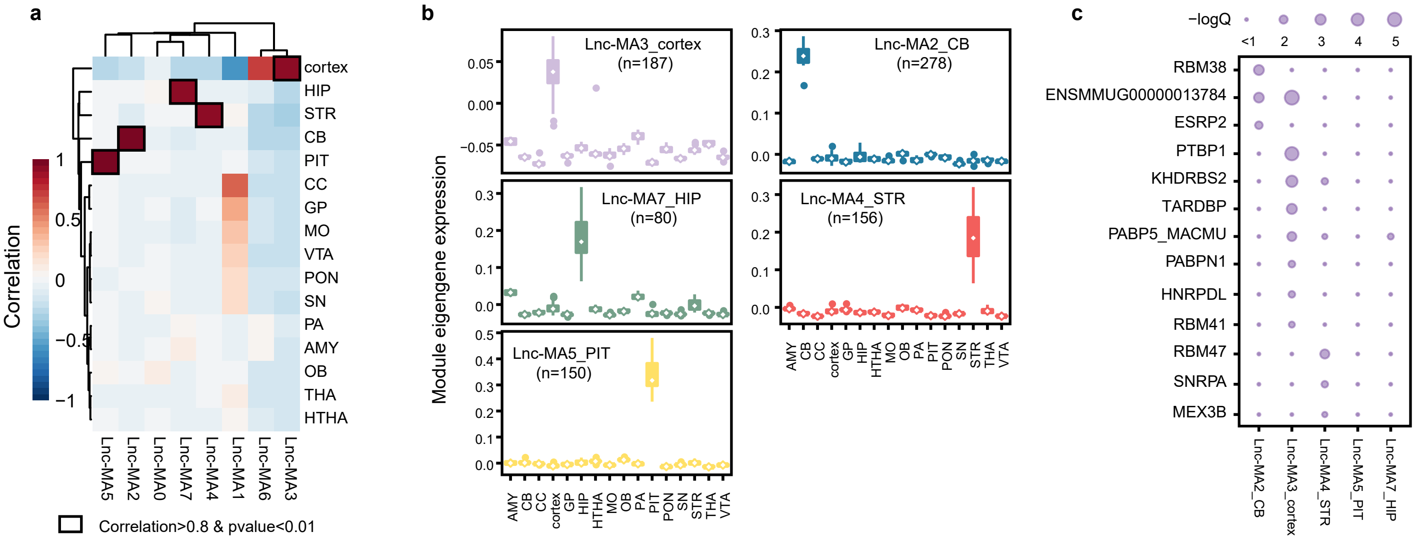

Supplementary Figure 6. Region-related novel lncRNA modules. (a) Heatmap showing the correlation between subregions and lncRNA modules. (b) Boxplot showing expression of the module eigengenes of the region-specific modules, the parenthesized number represented number of lncRNAs in each module. In boxplots, center point, box edges, whiskers and filled dot indicate the median, upper and lower quartiles (the 25th and 75th percentiles), 1.5× interquartile range and outlier, respectively. (c) Bubble plot showing the RBP-motif enrichment of module lncRNA sequences. Source data are provided as a Source Data file.

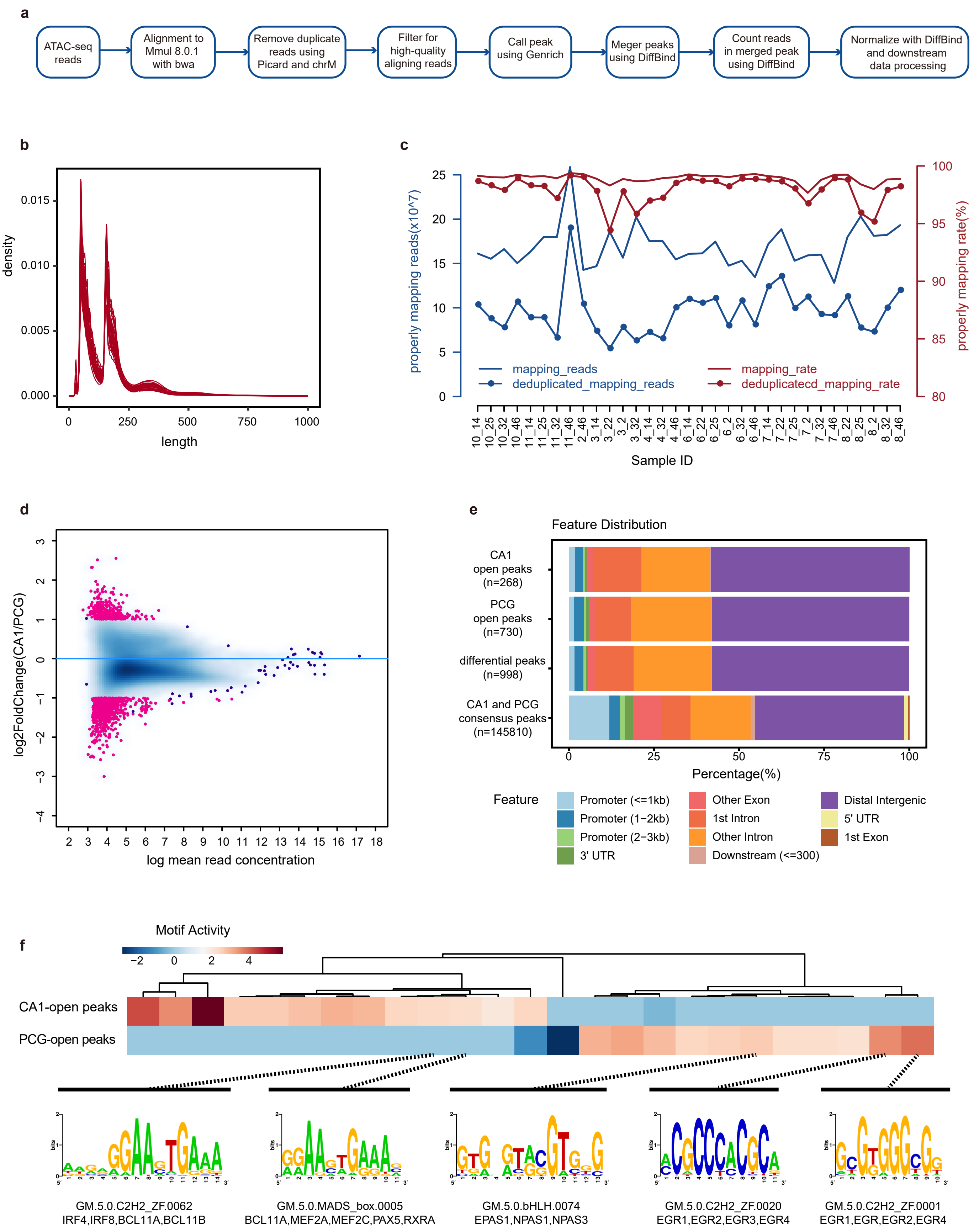

a
ATAC-seq reads
Alignment to Mmul 8.0.1 with bwa
Remove duplicate reads using Picard and chrM
Filter for high-quality aligning reads
Call peak using Genrich
Meger peaks using DiffBind
Count reads in merged peak using DiffBind
Normalize with DiffBind and downstream data processing
b
density
length
c
properly mapping reads(x10^7)
properly mapping rate(%)
mapping_reads
deduplicated_mapping_reads
mapping_rate
deduplicatecd_mapping_rate
Sample ID
d
log2FoldChange(CA1/PCG)
log mean read concentration
e
Feature Distribution
CA1 open peaks (n=268)
PCG open peaks (n=730)
differential peaks (n=998)
CA1 and PCG consensus peaks (n=145810)
Percentage(%)
Feature
Promoter (<=1kb)
Promoter (1−2kb)
Promoter (2−3kb)
3' UTR
Other Exon
1st Intron
Other Intron
Downstream (<=300)
Distal Intergenic
5' UTR
1st Exon
f
Motif Activity
CA1-open peaks
PCG-open peaks
GM.5.0.C2H2_ZF.0062
IRF4,IRF8,BCL11A,BCL11B
GM.5.0.MADS_box.0005
BCL11A,MEF2A,MEF2C,PAX5,RXRA
GM.5.0.bHLH.0074
EPAS1,NPAS1,NPAS3
GM.5.0.C2H2_ZF.0020
EGR1,EGR2,EGR3,EGR4
GM.5.0.C2H2_ZF.0001
EGR1,EGR,EGR2,EGR4

Supplementary Figure 7. Analysis framework and differential peaks of ATAC-seq. (a) The pipeline for ATAC-seq data processing. (b) The distribution of fragments length in ATAC-seq. (c) The plot showing the properly mapping reads and mapping rate of ATAC-seq data. (d) MA plot of CA1-PCG open peak comparison. Points in pink representing differential peaks (|log2FoldChange|>1, FDR<0.1). (e) Accumulated barplot showing the feature distribution of CA1-PCG differential peaks and CA1-PCG consensus peaks. (f) Heatmap visualizing the motif activity of the top 25 motifs (absolute motif activity >= 2). The color represents the reported motif activity, where the value corresponds to the log10 of the p-value of the rank aggregation. For high-ranking motifs (red) -log10(p-value) is shown, while for low ranking motifs (blue) log10(p-value) of the reversed ranking is shown. Sequence logos for motifs enriched in open chromatin regions were shown in the bottom. Source data are provided as a Source Data file.

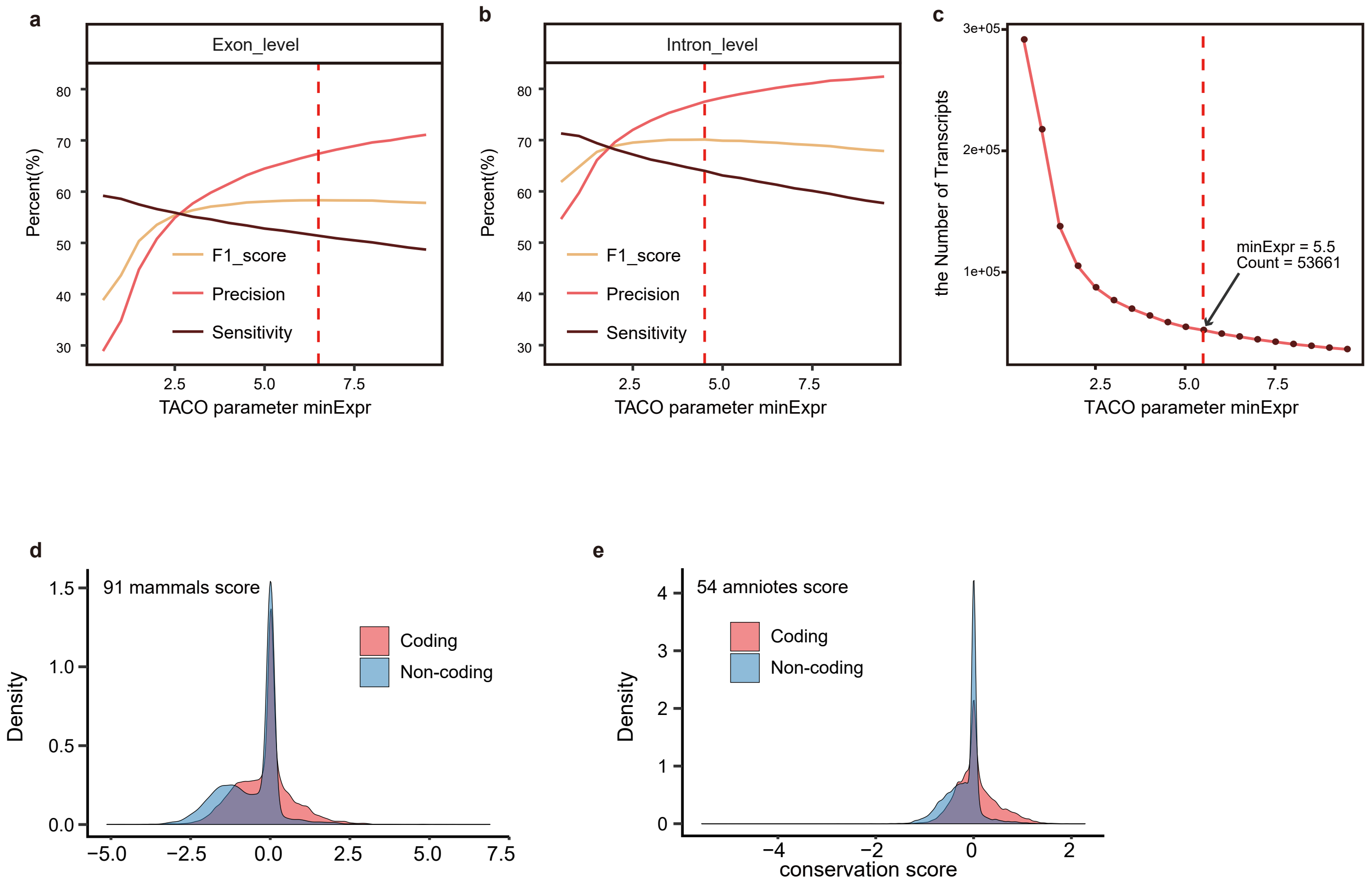

Supplementary Figure 8. Figures for supplementary notes. (a) The precision, sensitivity and F1 score under different minimum expression threshold at exon-level in TACO. (b) The precision, sensitivity and F1 score under different minimum expression threshold at intron level in TACO. (c) The number of transcripts under different minimum expression threshold. (d) **and** (e) Density plot showing the conservation score of novel coding and non-coding transcripts across 91 mammals and 54 amniotes, respectively. Source data are provided as a Source Data file.

Supplementary Tables

Supplementary Table 1. The information of sub-regions from Rhesus Macaque brain

ID	Sub-region	Region	Structure name	Region gruop
1	OB	frontal lobe	Olfactory bulb	OB
2	MFG	frontal lobe	middle frontal gyrus	cortex
3	IFG	frontal lobe	inferior frontal gyrus	cortex
4	SFG	frontal lobe	posterior superior frontal gyrus	cortex
5	ASFG	frontal lobe	anterior superior frontal gyrus	cortex
6	AASG	frontal lobe	anterior arcuate sulcus gyrus	cortex
7	vmPFC	frontal lobe	ventromedial prefrontal cortex	cortex
8	MOG	frontal lobe	medial orbital gyrus	cortex
9	INS	frontal lobe	insula	cortex
10	SPrCG	frontal lobe	superior precentral gyrus	cortex
11	IPrCG	frontal lobe	inferior precentral gyrus	cortex
12	SPCG	parietal lobe	superior postcentral gyrus	cortex
13	IPCG	parietal lobe	inferior postcentral gyrus	cortex
14	SPL	parietal lobe	superior parietal lobule	cortex
15	PSPG	parietal lobe	posterior superior parietal gyrus	cortex
16	IPS	parietal lobe	Intraparietal sulcus	cortex
17	AMG& PMG	parietal lobe	anterior/posterior marginal gyrus	cortex
18	ALG	parietal lobe	AG, angular gyrus	cortex
19	ATG	parietal lobe	annectant gyri, in macaque	cortex
20	CuG	parietal lobe	cuneus gyrus	cortex
21	aSOG	occipital lobe	anterior superior occipital gyrus	cortex
22	pSOG	occipital lobe	posterior superior occipital gyrus	cortex
23	IOG	occipital lobe	inferior occipital gyrus	cortex
24	OTG	temporal lobe	occipito-temporal gyrus	cortex
25	ITG	temporal lobe	inferior temporal gyrus	cortex
26	STG	temporal lobe	superior temporal gyrus	cortex
27	TPG	temporal lobe	temporal polar gyrus	cortex
28	PA	limbic system	entorhinal cortex & perirhinal cortex	PA
29	FG	limbic cortex	fusiform gyrus	cortex
30	HG	limbic cortex	hippocampal gyrus or Parahippocampal gyrus	cortex
31	LG	limbic cortex	lingual gyrus	cortex
32	PCG	limbic cortex	posterior cingular gyrus	cortex
33	ACG	limbic cortex	anterior cingular gyrus	cortex
34	CC	limbic system	corpus callosum	CC
35	THA	thalamus	thalamus	THA
36	HTHA	hypothalamus	hypothalamus	HTHA
37	MO	medulla oblongata	medulla oblongata	MO
38	PON	pons	pons	PON
39	CBC	cerebellum	cerebellar cortex	CB

40	CBV	cerebellum	cerebellum vermis	CB
41	VTA	Ventral tegmental area	Ventral tegmental area	VTA
42	SN	Substantia Nigra	Substantia Nigra	SN
43	MB	midbrain	midbrain	MB
44	AMY	amygdala	amygdala	AMY
45	DG	hippocampus	dentate gyrus	HIP
46	CA1	hippocampus	cornu Ammonis 1	HIP
47	CA3	hippocampus	cornu Ammonis 3	HIP
48	ACb	striatum	accumbens nucleus	STR
49	CN	striatum	caudate nucleus	STR
50	PUT	striatum	putamen	STR
51	GP	globus pallidus	globus pallidus	GP
52	PIT	pituitarium	pituitarium	PIT

Supplementary Table 2. Rhesus information

Sample	Sex	Age (yrs)	Age Stage	health condition
Rhesus_1	male	10	Mid	healthy
Rhesus_2	male	10	Mid	healthy
Rhesus_3	female	3.2	Young	healthy
Rhesus_4	female	10	Mid	healthy
Rhesus_5	male	4	Young	healthy
Rhesus_6	male	3.8	Young	healthy
Rhesus_7	female	5	Young	healthy
Rhesus_8	female	9.8	Mid	healthy

Supplementary Table 3. The differential expression genes between male and female

gene	gene_name	region	hyper	pvalue	log2FoldChange	Coefficient (Sex)	Coefficient (Age_Stage)
ENSMMUG00000030178	NA	PA	male	6.64E-14	3.404914574	2.590266281	-2.27726635
ENSMMUG00000004192	CFAP100	CC	male	9.99E-10	1.785163599	1.26623695	0.586114319
ENSMMUG00000016673	TTR	THA	male	2.73E-12	3.590201714	2.396075772	-0.612759464
ENSMMUG00000031684	FAM198A	HTHA	male	3.18E-08	1.48889278	1.099062611	0.321184962
ENSMMUG00000028680	ND3	HTHA	male	7.21E-08	6.781017734	4.732093555	-0.138109779
ENSMMUG00000016993	PKHD1	MO	male	5.63E-10	2.126481422	1.326871122	-0.471477688
ENSMMUG00000028680	ND3	MO	male	3.78E-07	6.731640491	4.541588858	-0.123669128
ENSMMUG00000004222	TBXAS1	MO	female	5.67E-18	-1.391516865	-1.105331547	-0.128454746
ENSMMUG00000011681	PTAFR	MO	female	1.71E-16	-1.224279439	-0.996650128	-0.264742155
ENSMMUG00000003191	KIRREL1	MO	female	1.89E-10	-1.072984879	-0.875468895	0.292259187
ENSMMUG00000013376	POU2F2	MO	female	4.87E-10	-1.278820777	-1.021831654	-0.17788787
ENSMMUG00000046537	NA	MO	female	7.27E-10	-1.001085959	-0.854983635	-0.528057163
ENSMMUG00000019159	PTK2B	MO	female	2.16E-09	-1.635561643	-1.25670072	0.575026151
ENSMMUG00000002419	TMEM156	MO	female	3.08E-09	-1.754256416	-1.40862234	-1.17714452
ENSMMUG00000000020	PTGDR2	MO	female	6.24E-09	-1.217156804	-0.965805117	-0.357607872
ENSMMUG00000004492	AIM2	MO	female	5.19E-08	-1.168274838	-0.960446775	-0.619988491
ENSMMUG00000012721	EFCAB12	MO	female	2.18E-07	-1.365728381	-1.08960206	0.016055827
ENSMMUG00000000170	SAMD9	MO	female	2.30E-07	-1.312403519	-1.046787098	-0.047250388
ENSMMUG00000044001	NA	MO	female	2.51E-07	-1.413287704	-1.122140712	-0.246851485
ENSMMUG00000010316	HERC5	MO	female	2.87E-07	-1.090426856	-0.92808919	-0.73774325
ENSMMUG00000042422	GNGT2	MO	female	4.56E-07	-1.87104357	-1.433327631	-0.555321396
ENSMMUG00000010647	NA	MO	female	5.70E-07	-1.405006825	-1.10980013	-0.273436857
ENSMMUG00000039909	NA	MO	female	8.48E-07	-3.322315993	-2.390773078	0.404461848
ENSMMUG00000032499	NA	MO	female	8.56E-07	-4.549479153	-3.305641379	1.150735565
ENSMMUG00000007080	HTR3A	MO	female	1.18E-06	-1.604559367	-1.325669621	3.258096464
ENSMMUG00000015250	CLEC4A	MO	female	1.19E-06	-1.072740347	-0.870590359	0.104521387
ENSMMUG00000042362	CLEC2B	MO	female	1.24E-06	-1.418846124	-1.115798427	-0.367747602
ENSMMUG00000016143	SPN	MO	female	1.59E-06	-1.09945697	-0.941392393	-0.723590354
ENSMMUG00000002267	LAMC3	MO	female	2.09E-06	-1.047074099	-0.860923364	-0.480764575
ENSMMUG00000028680	ND3	PON	male	2.77E-08	6.96137096	4.687404288	-0.553385127
ENSMMUG00000045895	NA	PON	female	8.32E-11	-5.794081547	-4.177924503	-0.810153984
ENSMMUG00000032499	NA	PON	female	2.42E-07	-4.482549789	-3.204227557	0.470285079
ENSMMUG00000014751	IL25	VTA	male	9.01E-10	1.079346337	0.750654799	-0.160504459
ENSMMUG00000028680	ND3	GP	male	3.32E-07	7.306738755	4.734050663	-0.550756584
ENSMMUG00000008316	NANOS3	GP	female	2.21E-07	-1.095976193	-0.996667037	0.446014728
ENSMMUG00000016294	SYCP2	PIT	male	7.37E-17	2.743004168	2.00617762	1.464250036
ENSMMUG00000048912	ECHDC3	PIT	male	3.19E-16	1.118684245	0.87250715	0.800903428
ENSMMUG00000020795	FGD3	PIT	male	2.46E-14	1.22331881	0.939657167	0.292037731
ENSMMUG00000020736	HACD1	PIT	male	8.67E-13	1.116221556	0.869374016	0.509185269
ENSMMUG00000012229	MYO3A	PIT	male	1.23E-12	1.21081971	0.960456575	0.747170195
ENSMMUG00000037552	LAIR2	PIT	male	8.18E-10	3.506757551	2.540781819	0.171009108
ENSMMUG00000005997	NA	PIT	female	1.26E-09	-1.25924769	-0.748299295	0.145955356

Supplementary Table 4. Human samples information from PsychEncode

Species	Brain	Region	NCXRegion	Age
Human	HSB122	AMY	AMY	1 Y
Human	HSB122	CBC	CBC	1 Y
Human	HSB122	HIP	HIP	1 Y
Human	HSB122	MD	THA	1 Y
Human	HSB122	A1C	cortex	1 Y
Human	HSB122	DFC	cortex	1 Y
Human	HSB122	IPC	cortex	1 Y
Human	HSB122	ITC	cortex	1 Y
Human	HSB122	M1C	cortex	1 Y
Human	HSB122	MFC	cortex	1 Y
Human	HSB122	OFC	cortex	1 Y
Human	HSB122	S1C	cortex	1 Y
Human	HSB122	STC	cortex	1 Y
Human	HSB122	V1C	cortex	1 Y
Human	HSB122	VFC	cortex	1 Y
Human	HSB122	STR	STR	1 Y
Human	HSB127	AMY	AMY	19 Y
Human	HSB127	CBC	CBC	19 Y
Human	HSB127	HIP	HIP	19 Y
Human	HSB127	MD	THA	19 Y
Human	HSB127	A1C	cortex	19 Y
Human	HSB127	DFC	cortex	19 Y
Human	HSB127	IPC	cortex	19 Y
Human	HSB127	ITC	cortex	19 Y
Human	HSB127	M1C	cortex	19 Y
Human	HSB127	MFC	cortex	19 Y
Human	HSB127	OFC	cortex	19 Y
Human	HSB127	S1C	cortex	19 Y
Human	HSB127	STC	cortex	19 Y
Human	HSB127	V1C	cortex	19 Y
Human	HSB127	VFC	cortex	19 Y
Human	HSB127	STR	STR	19 Y
Human	HSB130	AMY	AMY	21 Y
Human	HSB130	CBC	CBC	21 Y
Human	HSB130	HIP	HIP	21 Y
Human	HSB130	MD	THA	21 Y
Human	HSB130	A1C	cortex	21 Y
Human	HSB130	DFC	cortex	21 Y
Human	HSB130	IPC	cortex	21 Y
Human	HSB130	ITC	cortex	21 Y
Human	HSB130	M1C	cortex	21 Y
Human	HSB130	MFC	cortex	21 Y
Human	HSB130	OFC	cortex	21 Y
Human	HSB130	S1C	cortex	21 Y
Human	HSB130	STC	cortex	21 Y
Human	HSB130	V1C	cortex	21 Y

Human	HSB130	VFC	cortex	21 Y
Human	HSB130	STR	STR	21 Y
Human	HSB145	AMY	AMY	36 Y
Human	HSB145	CBC	CBC	36 Y
Human	HSB145	HIP	HIP	36 Y
Human	HSB145	MD	THA	36 Y
Human	HSB145	A1C	cortex	36 Y
Human	HSB145	DFC	cortex	36 Y
Human	HSB145	IPC	cortex	36 Y
Human	HSB145	ITC	cortex	36 Y
Human	HSB145	M1C	cortex	36 Y
Human	HSB145	MFC	cortex	36 Y
Human	HSB145	OFC	cortex	36 Y
Human	HSB145	S1C	cortex	36 Y
Human	HSB145	STC	cortex	36 Y
Human	HSB145	V1C	cortex	36 Y
Human	HSB145	VFC	cortex	36 Y
Human	HSB145	STR	STR	36 Y
Human	HSB123	AMY	AMY	37 Y
Human	HSB123	CBC	CBC	37 Y
Human	HSB123	HIP	HIP	37 Y
Human	HSB123	MD	THA	37 Y
Human	HSB123	A1C	cortex	37 Y
Human	HSB123	DFC	cortex	37 Y
Human	HSB123	IPC	cortex	37 Y
Human	HSB123	ITC	cortex	37 Y
Human	HSB123	M1C	cortex	37 Y
Human	HSB123	MFC	cortex	37 Y
Human	HSB123	OFC	cortex	37 Y
Human	HSB123	S1C	cortex	37 Y
Human	HSB123	STC	cortex	37 Y
Human	HSB123	V1C	cortex	37 Y
Human	HSB123	VFC	cortex	37 Y
Human	HSB123	STR	STR	37 Y

Supplementary Table 5. Correspondence between human and rhesus macaque in cortex region

human region	human structure name	Rhesus region	Rhesus structure name
A1C	primary auditory cortex (core)	STG	superior temporal gyrus
STC	posterior (caudal) superior temporal cortex (area 22c)		
DFC	dorsolateral prefrontal cortex	MFG	middle frontal gyrus
IPC	posteroventral (inferior) parietal cortex	AMG&PMG	anterior/posterior marginal gyrus
		ALG	AG, angular gyrus
ITC	inferolateral temporal cortex (area TEv, area 20)	ITG	ITG, inferior temporal gyrus
M1C	primary motor cortex (area M1, area 4)	SPrCG/IPrCG	superior/inferior precentral gyrus
MFC	anterior (rostral) cingulate (medial prefrontal) cortex	ACG	anterior cingular gyrus
OFC	orbital frontal cortex	MOG	Medial orbital gyrus
S1C	primary somatosensory cortex (area S1, areas 3,1,2)	SPCG	superior postcentral gyrus
		IPCG	inferior postcentral gyrus
V1C	primary visual cortex (striate cortex, area V1/17)	aSOG/pSOG	posterior superior occipital gyrus
VFC	ventrolateral prefrontal cortex	IFG	inferior frontal gyrus

Supplementary Table 6. The enrichment function of WGCNA modules

Domain	Term ID	Pvalue	Term name
**M2-PIT**			
keg	KEGG:04141	2.68E-08	Protein processing in endoplasmic reticulum
CC	GO:0005783	7.06E-05	endoplasmic reticulum
CC	GO:0012505	9.56E-05	endomembrane system
CC	GO:0031984	0.000475	organelle subcompartment
CC	GO:0005576	0.00133	extracellular region
CC	GO:0044431	0.00228	Golgi apparatus part
CC	GO:0030990	0.0038	intraciliary transport particle
CC	GO:0044421	0.00498	extracellular region part
CC	GO:0005929	0.0083	cilium
CC	GO:0005793	0.00931	endoplasmic reticulum-Golgi intermediate compartment
CC	GO:0098791	0.00985	Golgi subcompartment
BP	GO:0060271	0.0105	cilium assembly
BP	GO:0044782	0.013	cilium organization
CC	GO:0030660	0.0143	Golgi-associated vesicle membrane
hp	HP:0000682	0.0234	Abnormality of dental enamel
CC	GO:0005615	0.0241	extracellular space
CC	GO:0070160	0.0259	occluding junction
hp	HP:3000050	0.0265	Abnormality of odontoid tissue
CC	GO:0031982	0.033	vesicle
CC	GO:0031012	0.0361	extracellular matrix
CC	GO:0005798	0.0369	Golgi-associated vesicle
CC	GO:0000139	0.0379	Golgi membrane
**M6-STR**			
BP	GO:0007188	0.00069	adenylate cyclase-modulating G-protein coupled receptor signaling pathway
BP	GO:0007189	0.00133	adenylate cyclase-activating G-protein coupled receptor signaling pathway
BP	GO:0007187	0.00156	G-protein coupled receptor signaling pathway, coupled to cyclic nucleotide second messenger
keg	KEGG:04024	0.00174	cAMP signaling pathway
BP	GO:0019933	0.00287	cAMP-mediated signaling
BP	GO:0050801	0.00402	ion homeostasis
hp	HP:0001013	0.00548	Eruptive xanthomas
BP	GO:0019935	0.00826	cyclic-nucleotide-mediated signaling
CC	GO:0005886	0.00978	plasma membrane
BP	GO:0040013	0.0123	negative regulation of locomotion
BP	GO:0030336	0.0134	negative regulation of cell migration
BP	GO:0051271	0.0179	negative regulation of cellular component movement
keg	KEGG:00230	0.0186	Purine metabolism
BP	GO:0019932	0.0188	second-messenger-mediated signaling
CC	GO:0071944	0.019	cell periphery
BP	GO:0098771	0.0241	inorganic ion homeostasis
BP	GO:0007154	0.0246	cell communication
BP	GO:0048878	0.0281	chemical homeostasis
BP	GO:2000146	0.0304	negative regulation of cell motility

BP	GO:0007186	0.0311	G-protein coupled receptor signaling pathway
BP	GO:0023052	0.0335	signaling
BP	GO:0006873	0.0466	cellular ion homeostasis
BP	GO:0014075	0.0495	response to amine
BP	GO:0001975	0.0495	response to amphetamine
**M7-CB**			
keg	KEGG:04360	0.0254	Axon guidance
**M10-cortex2**			
BP	GO:0045595	3.38E-08	regulation of cell differentiation
BP	GO:0050793	3.81E-06	regulation of developmental process
BP	GO:2000026	7.52E-05	regulation of multicellular organismal development
BP	GO:0051239	0.000245	regulation of multicellular organismal process
BP	GO:0051960	0.000897	regulation of nervous system development
BP	GO:0010975	0.00119	regulation of neuron projection development
BP	GO:0050767	0.00142	regulation of neurogenesis
BP	GO:0023052	0.00261	signaling
BP	GO:0045664	0.0037	regulation of neuron differentiation
BP	GO:0050789	0.00454	regulation of biological process
BP	GO:0060284	0.00488	regulation of cell development
BP	GO:0050896	0.00541	response to stimulus
BP	GO:0050808	0.00792	synapse organization
BP	GO:0048869	0.0119	cellular developmental process
keg	KEGG:04913	0.0129	Ovarian steroidogenesis
BP	GO:0007154	0.013	cell communication
BP	GO:0030154	0.0135	cell differentiation
BP	GO:0048518	0.0157	positive regulation of biological process
BP	GO:0045597	0.0272	positive regulation of cell differentiation
BP	GO:0065007	0.0382	biological regulation
BP	GO:0051094	0.0425	positive regulation of developmental process
BP	GO:0065009	0.043	regulation of molecular function
**M11-OB**			
CC	GO:0031012	1.87E-09	extracellular matrix
CC	GO:0005576	2.39E-07	extracellular region
BP	GO:0032501	4.26E-07	multicellular organismal process
CC	GO:0044421	2.44E-05	extracellular region part
CC	GO:0005615	3.81E-05	extracellular space
keg	KEGG:05144	4.92E-05	Malaria
CC	GO:0005581	0.000202	collagen trimer
BP	GO:0009653	0.000393	anatomical structure morphogenesis
BP	GO:0023052	0.000799	signaling
BP	GO:0070208	0.000975	protein heterotrimerization
BP	GO:0007275	0.00116	multicellular organism development
BP	GO:0048731	0.0012	system development
BP	GO:0007154	0.00133	cell communication
BP	GO:0051239	0.00168	regulation of multicellular organismal process
BP	GO:0050896	0.00223	response to stimulus
BP	GO:0030198	0.00228	extracellular matrix organization
BP	GO:0003008	0.00247	system process
BP	GO:0009887	0.00292	animal organ morphogenesis

BP	GO:0072359	0.00422	circulatory system development
BP	GO:0043062	0.00547	extracellular structure organization
BP	GO:0009605	0.00582	response to external stimulus
hp	HP:0008628	0.00624	Abnormality of the stapes
BP	GO:0030199	0.00715	collagen fibril organization
BP	GO:0016477	0.00763	cell migration
BP	GO:0008217	0.00948	regulation of blood pressure
BP	GO:0001503	0.0106	ossification
BP	GO:0048513	0.0109	animal organ development
BP	GO:0060415	0.0116	muscle tissue morphogenesis
hp	HP:0004219	0.0117	Abnormality of the middle phalanx of the 5th finger
hp	HP:0008873	0.0119	Disproportionate short-limb short stature
BP	GO:0008015	0.0172	blood circulation
hp	HP:0006460	0.0173	Increased laxity of ankles
BP	GO:0007166	0.0211	cell surface receptor signaling pathway
BP	GO:0003013	0.0212	circulatory system process
BP	GO:0044057	0.0212	regulation of system process
BP	GO:0048644	0.0222	muscle organ morphogenesis
BP	GO:0051674	0.025	localization of cell
BP	GO:0048870	0.025	cell motility
BP	GO:0001501	0.0264	skeletal system development
CC	GO:0098643	0.0271	banded collagen fibril
CC	GO:0005583	0.0271	fibrillar collagen trimer
BP	GO:0051716	0.0275	cellular response to stimulus
keg	KEGG:04974	0.028	Protein digestion and absorption
BP	GO:0009607	0.0307	response to biotic stimulus
BP	GO:0007165	0.0319	signal transduction
BP	GO:0003073	0.0346	regulation of systemic arterial blood pressure
hp	HP:0000592	0.0346	Blue sclerae
CC	GO:0044420	0.0481	extracellular matrix component
hp	HP:0004452	0.0494	Abnormality of the middle ear ossicles
CC	GO:0062023	0.0499	collagen-containing extracellular matrix
**M25-HIP**			
CC	GO:0016021	0.00208	integral component of membrane
CC	GO:0031224	0.00329	intrinsic component of membrane
BP	GO:0048589	0.00585	developmental growth
BP	GO:0030154	0.027	cell differentiation
BP	GO:0097475	0.0277	motor neuron migration
MF	GO:0004983	0.0277	neuropeptide Y receptor activity
CC	GO:0005887	0.028	integral component of plasma membrane
MF	GO:0004888	0.032	transmembrane signaling receptor activity
BP	GO:0040007	0.0392	growth
BP	GO:0032502	0.0494	developmental process
BP	GO:0048869	0.0496	cellular developmental process
**M28-THA**			
keg	KEGG:04974	0.00408	Protein digestion and absorption
BP	GO:0051239	0.00808	regulation of multicellular organismal process
BP	GO:0042391	0.0129	regulation of membrane potential
MF	GO:0030545	0.0221	receptor regulator activity

BP	GO:0010469	0.0285	regulation of signaling receptor activity
BP	GO:0048513	0.0496	animal organ development
**M36-SN**			
hp	HP:0002451	1.48E-05	Limb dystonia
BP	GO:0009636	4.32E-05	response to toxic substance
BP	GO:0042416	5.86E-05	dopamine biosynthetic process
BP	GO:0009713	0.000543	catechol-containing compound biosynthetic process
BP	GO:0042423	0.000543	catecholamine biosynthetic process
hp	HP:0003785	0.00128	Decreased CSF homovanillic acid
BP	GO:0042417	0.00767	dopamine metabolic process
BP	GO:0046189	0.00943	phenol-containing compound biosynthetic process
BP	GO:0009712	0.0165	catechol-containing compound metabolic process
BP	GO:0006584	0.0165	catecholamine metabolic process
BP	GO:0018958	0.0175	phenol-containing compound metabolic process
**M45-HTHA**			
BP	GO:0007218	0.00068	neuropeptide signaling pathway
MF	GO:0005179	0.0013	hormone activity
BP	GO:0007186	0.00229	G-protein coupled receptor signaling pathway
MF	GO:0071855	0.0441	neuropeptide receptor binding
BP	GO:0043396	0.0498	corticotropin-releasing hormone secretion
BP	GO:0043397	0.0498	regulation of corticotropin-releasing hormone secretion

Supplementary Table 7. The sub-region specific genes within CB

Gene ID	Gene name	Hyper-Region	CBC (mean)	CBV (mean)	pvalue	padj
ENSMMUG00000003276	LAMP5	CBC	143.6647832	55.95880983	8.48E-17	2.70E-13
ENSMMUG00000000223	HTR1E	CBC	75.82244728	30.96530297	2.44E-14	4.87E-11
ENSMMUG00000041480	IGFBP5	CBC	3378.487999	1677.70005	4.32E-13	5.29E-10
ENSMMUG00000016887	WIF1	CBC	185.9679354	71.36356659	3.58E-11	2.59E-08
ENSMMUG00000011579	FAM19A2	CBC	275.8813187	135.5346931	1.03E-10	6.83E-08
ENSMMUG00000007176	DYSF	CBC	93.4407657	46.19083105	1.15E-10	7.33E-08
ENSMMUG00000006910	UGT3A2	CBC	59.50442692	22.38676349	4.05E-09	1.79E-06
ENSMMUG00000013649	MYOC	CBC	198.5183899	120.5608806	5.61E-09	2.35E-06
ENSMMUG00000015604	MYBPC3	CBC	68.06447086	30.58625481	1.26E-08	4.91E-06
ENSMMUG00000023324	MUM1L1	CBC	107.5812351	37.50694887	2.40E-08	8.89E-06
ENSMMUG00000020426	SERPINA1	CBC	308.4605948	140.046532	6.89E-08	2.39E-05
ENSMMUG00000017249	EPN3	CBC	85.43333734	40.5837915	4.90E-07	0.000139437
ENSMMUG00000014725	NA	CBC	35.99325044	11.66222855	2.38E-06	0.000520274
ENSMMUG00000021977	PI16	CBC	43.78626419	13.27445658	2.62E-06	0.000563598
ENSMMUG00000023190	TCIRG1	CBC	260.9400642	134.6723748	3.20E-06	0.000654186
ENSMMUG00000011763	AQP1	CBC	120.4602827	56.9460512	4.70E-06	0.000886059
ENSMMUG00000015572	GSG1L	CBC	52.10141908	22.08637878	5.73E-06	0.001024887
ENSMMUG00000000882	SPAG6	CBC	25.47064638	8.607745264	9.50E-06	0.001514027
ENSMMUG00000045540	NA	CBC	10.41267696	2.248846951	1.71E-05	0.002383855
ENSMMUG00000008855	TRABD2A	CBC	22.09234459	5.518468697	1.76E-05	0.002416017
ENSMMUG00000041594	PRSS56	CBC	29.47147391	12.86466603	2.32E-05	0.002908222
ENSMMUG00000007555	SEMA3A	CBC	22.16133159	3.207728729	2.69E-05	0.003271759
ENSMMUG00000018652	HGF	CBC	31.69051518	12.66147524	2.82E-05	0.003353578
ENSMMUG00000000540	KCNG4	CBC	22.01870452	9.568108799	7.87E-05	0.007839212
ENSMMUG00000023057	IFNLR1	CBC	29.63464604	14.40016927	0.000139675	0.012362003
ENSMMUG00000044692	C1orf53	CBC	9.979720817	3.302212766	0.000490142	0.036150257
ENSMMUG00000017711	LZTS1	CBC	22.71564077	6.251820302	0.000506961	0.037218389
ENSMMUG00000002234	FBLN7	CBC	17.03459174	7.40551142	0.000714522	0.048233229
ENSMMUG00000038006	OTX2	CBV	27.87624481	536.9203845	1.84E-56	2.93E-52
ENSMMUG00000018511	CDH8	CBV	17.13305299	284.3537075	2.47E-41	1.97E-37
ENSMMUG00000021914	SPON1	CBV	329.0358077	832.7044567	2.84E-21	1.51E-17
ENSMMUG00000009091	MTTP	CBV	87.90042181	254.6708451	3.14E-15	7.15E-12
ENSMMUG00000014646	NTF3	CBV	125.7640223	251.4165091	1.19E-13	1.89E-10
ENSMMUG00000011559	SLC6A7	CBV	153.9880673	325.8549355	4.27E-13	5.29E-10
ENSMMUG00000015685	ZNF804B	CBV	185.916796	368.8010299	9.01E-12	7.56E-09
ENSMMUG00000014823	TAC1	CBV	17.45236575	49.276995	5.30E-11	3.67E-08
ENSMMUG00000048564	NA	CBV	15.62589566	41.34677178	6.63E-10	3.30E-07
ENSMMUG00000038637	NA	CBV	9.357295872	46.92575763	3.34E-09	1.57E-06
ENSMMUG00000017302	HPCA	CBV	190.8444948	544.2445513	3.75E-09	1.71E-06
ENSMMUG00000017301	TMEM54	CBV	174.2859135	470.1552399	8.37E-09	3.42E-06
ENSMMUG00000020406	GCNT1	CBV	45.62157757	95.380105	2.13E-07	6.79E-05
ENSMMUG00000017177	10-Sep	CBV	80.95244096	180.0917812	1.51E-06	0.000349759
ENSMMUG00000042253	NA	CBV	3.936889955	13.78445526	8.68E-06	0.00141072
ENSMMUG00000017179	SOWAHC	CBV	9.846743732	26.05511135	1.66E-05	0.002338516
ENSMMUG00000049079	NA	CBV	6.528431265	18.9522175	4.76E-05	0.005051034
ENSMMUG00000007730	TPH2	CBV	7.817359025	25.07834917	7.49E-05	0.0075977

Supplementary Table 8. Shared gene name between novel transcripts and reference

Transcripts ID	Reference transcripts ID	similarity	Gene name
TU247	ENSMMUT00000016693	97.386	ENO1
TU26932	ENSMMUT00000022598	82.127	ZNF816
TU3583	ENSMMUT00000071979	97.802	ARF1
TU40311	ENSMMUT00000060772	98.706	MAD2L1
TU4889	ENSMMUT00000003601	97.717	TOMM20
TU52357	ENSMMUT00000029021	95.299	IGBP1
TU5354	ENSMMUT00000030554	99.295	VAPB
TU6586	ENSMMUT00000043652	97.458	MRPL40

Supplementary Table 9. ATAC-seq mapping information

sample_id	reads		mapping rate(%)	
	origin	deduplicated	origin	deduplicated
10_14	161255962	103505179	99.2	98.68
10_25	155329129	87963974	99.03	98.30
10_32	166206791	77884533	99.00	97.89
10_46	150420871	106803483	99.23	98.9
11_14	163370196	89238487	99.07	98.31
11_25	179861078	89064150	99.12	98.24
11_32	179834989	66336473	98.94	97.18
11_46	258753685	189993517	99.38	99.16
2_46	142769875	104132699	99.29	99.03
3_14	146971378	73938591	98.89	97.82
3_22	185826542	54357422	98.30	94.42
3_2	156530025	78199760	98.87	97.77
3_32	202499717	62802316	98.67	95.83
4_14	175237338	72531591	98.73	96.98
4_32	175356236	65364033	98.94	97.21
4_46	154556364	100233036	99.03	98.51
6_14	160906908	110044106	99.27	98.94
6_22	161409479	105341167	99.14	98.69
6_25	174711794	110564134	99.15	98.67
6_2	147512791	79957994	99.0	98.22
6_32	153079593	108065493	99.21	98.88
6_46	134649333	81224428	99.30	98.85
7_14	171711249	123782715	99.1	98.79
7_22	188674075	135799097	99.02	98.65
7_25	153075044	99548055	98.71	98.0
7_2	159160912	112173607	97.67	96.72
7_32	159975640	92787746	98.79	97.94
7_46	128150883	91550332	99.23	98.93
8_22	179619455	112553512	99.24	98.79
8_25	203104536	77331659	98.41	95.93
8_2	181222378	73058350	97.99	95.15
8_32	182197207	99989208	98.83	97.89
8_46	193220130	120109143	98.88	98.21
