# Supplementary material for: Transcriptomic and open chromatin atlas of high-resolution anatomical regions in the rhesus macaque brain
Source: Nat Commun. 2020 Jan 24;11:474. doi: 10.1038/s41467-020-14368-z (PMC6981234; doi:10.1038/s41467-020-14368-z)
Supplement: Supplementary file 3 — Description of Additional Supplementary Files [file 41467_2020_14368_MOESM3_ESM.docx]

**Description of Additional Supplementary Files**

**Supplementary Data 1.** The quality of Rhesus macaque RNA-seq

**Supplementary Data 2.** The differential expression genes between Mid and Young

**Supplementary Data 3.** Species-specific genes between human and rhesus macaque

**Supplementary Data 4.** The gene list of region-related modules

**Supplementary Data 5.** The gene list of lobe-specific modules in cortex

**Supplementary Data 6.** The sub-region specific genes within STR

**Supplementary Data 7.** The sub-region specific genes within HIP

**Supplementary Data 8.** The list of novel coding transcripts

**Supplementary Data 9.** The information of LncRNAs

**Supplementary Data 10.** The list of region-specific modules of novel lncRNA

**Supplementary Data 11.** The links between CA1 open peaks and genes

**Supplementary Data 12.** The links between PCG open peaks and genes
