# Supplementary material for: Transcriptomic and open chromatin atlas of high-resolution anatomical regions in the rhesus macaque brain
Source: Nat Commun. 2020 Jan 24;11:474. doi: 10.1038/s41467-020-14368-z (PMC6981234; doi:10.1038/s41467-020-14368-z)
Supplement: Supplementary file 16 — Reporting Summary [file 41467_2020_14368_MOESM16_ESM.pdf]

nature research

Corresponding author(s): Yong Fan, Dan Xie, Yang Yu

Last updated by author(s): Jan 2, 2020

Reporting Summary

Nature Research wishes to improve the reproducibility of the work that we publish. This form provides structure for consistency and transparency in reporting. For further information on Nature Research policies, see Authors & Referees and the Editorial Policy Checklist .

Statistics

For all statistical analyses, confirm that the following items are present in the figure legend, table legend, main text, or Methods section.

n/a	Confirmed	
☐	☒	The exact sample size (*n*) for each experimental group/condition, given as a discrete number and unit of measurement
☐	☒	A statement on whether measurements were taken from distinct samples or whether the same sample was measured repeatedly
☐	☒	The statistical test(s) used AND whether they are one- or two-sided<br>*Only common tests should be described solely by name; describe more complex techniques in the Methods section.*
☐	☒	A description of all covariates tested
☐	☒	A description of any assumptions or corrections, such as tests of normality and adjustment for multiple comparisons
☐	☒	A full description of the statistical parameters including central tendency (e.g. means) or other basic estimates (e.g. regression coefficient) AND variation (e.g. standard deviation) or associated estimates of uncertainty (e.g. confidence intervals)
☐	☒	For null hypothesis testing, the test statistic (e.g. *F*, *t*, *r*) with confidence intervals, effect sizes, degrees of freedom and *P* value noted<br>*Give P values as exact values whenever suitable.*
☒	☐	For Bayesian analysis, information on the choice of priors and Markov chain Monte Carlo settings
☐	☒	For hierarchical and complex designs, identification of the appropriate level for tests and full reporting of outcomes
☐	☒	Estimates of effect sizes (e.g. Cohen's *d*, Pearson's *r*), indicating how they were calculated

Our web collection on statistics for biologists contains articles on many of the points above.

Software and code

Policy information about availability of computer code

Data collection	Eight healthy rhesus monkeys with balanced age and sex from a single source were used in this study. For each individual, the brain was dissected into 52 regions. RNA-seq and ATAC-seq were performed.
Data analysis	RNA-seq data was processed using HISAT2(v2.0.5), HTSeq (v0.6.1), DESeq2 (v1.18.1), WGCNA(v1.63), TACO (v0.7.3) and CPAT(v1.2.4)<br>ATAC-seq data was processed using BWA(v0.7.12), MACS2 (v2.1.0.20151222), bedtools(v2.25.0) and CHIPseeker (v1.14.2).

For manuscripts utilizing custom algorithms or software that are central to the research but not yet described in published literature, software must be made available to editors/reviewers. We strongly encourage code deposition in a community repository (e.g. GitHub). See the Nature Research guidelines for submitting code & software for further information.

Data

Policy information about availability of data

All manuscripts must include a data availability statement. This statement should provide the following information, where applicable:

- Accession codes, unique identifiers, or web links for publicly available datasets
- A list of figures that have associated raw data
- A description of any restrictions on data availability

Sequencing data are available at the GEO under accession GSE128537 (https://www.ncbi.nlm.nih.gov/geo/query/acc.cgi?acc=GSE128537&token=olyrwqeybluxrgp)

Field-specific reporting

Please select the one below that is the best fit for your research. If you are not sure, read the appropriate sections before making your selection.

Life sciences study design

All studies must disclose on these points even when the disclosure is negative.

Sample size	no sample-size calculation was performed, the sample sizes (8 individuals and 52 regions) were chosen based on previously published studies on rhesus macaque brain.
Data exclusions	Samples from the midbrain (n=8) were excluded from downstream analysis except for de novo transcriptome assembly, due to its mixed composition.
Replication	No replication.
Randomization	No randomization performed.
Blinding	This study involved no blinding since sequencing data were quantitative and the results were independent of blinding.

Reporting for specific materials, systems and methods

We require information from authors about some types of materials, experimental systems and methods used in many studies. Here, indicate whether each material, system or method listed is relevant to your study. If you are not sure if a list item applies to your research, read the appropriate section before selecting a response.

Materials & experimental systems

n/a	Involved in the study
☒	☐ Antibodies
☒	☐ Eukaryotic cell lines
☒	☐ Palaeontology
☐	☒ Animals and other organisms
☒	☐ Human research participants
☒	☐ Clinical data

Methods

n/a	Involved in the study
☒	☐ ChIP-seq
☒	☐ Flow cytometry
☒	☐ MRI-based neuroimaging

Animals and other organisms

Policy information about studies involving animals; ARRIVE guidelines recommended for reporting animal research

Laboratory animals	Eight healthy rhesus monkeys with balanced age (3-10 years old) and sex from a single source were used in this study
Wild animals	This study did not involve wild animals.
Field-collected samples	This study did not involve samples collected from the field.
Ethics oversight	The procedure of execution and brain harvest was standardized and in accordance with the Animal Ethics Procedures and Guidelines of the People's Republic of China.

Note that full information on the approval of the study protocol must also be provided in the manuscript.
